# First-Order Dynamic Modeling and Control of Soft Robots

**DOI:** 10.3389/frobt.2020.00095

**Published:** 2020-07-21

**Authors:** Thomas George Thuruthel, Federico Renda, Fumiya Iida

**Affiliations:** ^1^Bio-Inspired Robotics Lab, Department of Engineering, University of Cambridge, Cambridge, United Kingdom; ^2^Khalifa University Center for Autonomous Robotic Systems, Khalifa University of Science and Technology, Abu Dhabi, United Arab Emirates

**Keywords:** soft robotics, control, machine learning, dynamic modeling, first-order dynamics, model reduction

## Abstract

Modeling of soft robots is typically performed at the static level or at a second-order fully dynamic level. Controllers developed upon these models have several advantages and disadvantages. Static controllers, based on the kinematic relations tend to be the easiest to develop, but by sacrificing accuracy, efficiency and the natural dynamics. Controllers developed using second-order dynamic models tend to be computationally expensive, but allow optimal control. Here we propose that the dynamic model of a soft robot can be reduced to first-order dynamical equation owing to their high damping and low inertial properties, as typically observed in nature, with minimal loss in accuracy. This paper investigates the validity of this assumption and the advantages it provides to the modeling and control of soft robots. Our results demonstrate that this model approximation is a powerful tool for developing closed-loop task-space dynamic controllers for soft robots by simplifying the planning and sensory feedback process with minimal effects on the controller accuracy.

## 1. Introduction

Soft robotic technologies are becoming increasingly prevalent in the design and development of robots (Kim et al., 2013). Subsequently, there has been growing interests in the modeling and control of soft bodied systems, Unlike robots designed with rigid components, soft robotic systems present novel challenges and opportunities in developing their controllers (George Thuruthel et al., 2018).

The most common modeling and control strategy for soft robots are based on steady-state models, which, under the steady-state assumption, can be equated to the kinematic model (George Thuruthel et al., 2018). For cylindrically-shaped soft robots, this leads to the popular constant curvature model (Webster and Jones, 2010). For other shapes, geometrically exact models or Finite Element Method have been proposed (Trivedi et al., 2008; Renda et al., 2012; Duriez, 2013; Gong et al., 2018). Machine learning techniques can also be used to develop such mappings in a model-free manner (Giorelli et al., 2013; George Thuruthel et al., 2017; Jiang et al., 2017). Refer to Sadati et al. (2017) for a detailed comparison into multiple static modeling techniques. Due to their steady-state assumptions, such controllers will, however, be limited in their reachability, efficiency, and speed. Therefore, controllers developed from dynamic models are much more desirable.

A popular method for developing dynamic models for soft robots is based on the cosserat-rod mechanics. Such models have been extensively used for soft robotic manipulators driven by tendon actuation (Rucker and Webster, 2011; Renda et al., 2014, 2018). For fluidic actuation, other models have been adopted (Marchese et al., 2016; Della Santina et al., 2019). Hybrid models based on lumped mass systems also looks promising for general soft robots (Sadati et al., 2019). However, all these models will be more computationally intensive than their static counterparts. Learning-based models are a promising alternative in such cases (Thuruthel et al., 2017; Gillespie et al., 2018). Nevertheless, deriving control strategies from dynamic models, in general, introduces additional complexities in motion planning. For an alternate approach that introduces a control-oriented modeling of soft robots, the readers are suggested to look into Della Santina and Rus (2019).

Unlike static controllers, developing fully-dynamic controllers would involve a planning stage. Typically, this has to be performed using some optimization techniques irrespective of the modeling strategy. A good example is the use of trajectory optimization for the control of a soft robotic manipulator using a model-based (Marchese et al., 2016) and model-free method (Thuruthel et al., 2017). This process is time consuming and hence debilitating for closed-loop dynamic control. For fully-actuated soft robots, closed-loop dynamic controllers can be developed in the configuration space (Della Santina et al., 2018). For task-space closed loop control, model-based reinforcement learning is the only viable solution till now, however, they tend to be highly task specific and time consuming (Thuruthel et al., 2018).

This article investigates the viability of a first-order dynamic model for soft robots. It must be noted that unlike state-space dimensionality reduction methods (Thieffry et al., 2018), we are reducing the temporal dimensionality of the dynamic model. Such a model reduction is based on the hypothesis that soft robots typically have high damping and low inertial properties. This makes it possible to approximate the second-order dynamic model to a first-order dynamic model by ignoring the inertial terms (Strogatz, 2001). Even in nature, the ubiquitous muscle dynamics can be modeled as a first-order dynamical system (Zajac, 1989). This model-order reduction provides two advantages. First, first-order dynamical systems are computationally cheaper than second-order dynamical systems. Second, it opens the possibility to develop novel closed-loop control strategies using the reduced-order state feedback. Here, we show the direct learning of the operational space dynamics of the first-order dynamic model. Due to simplifying step, controllers can be easily developed using machine learning and a simple path planning algorithm. Moreover, the sensory requirements for closed-loop dynamic control is reduced because of the simplification.

We investigate the viability of this simplifying assumption using extensive simulation studies. First, we present the theoretical reasoning behind the first-order assumption and its corresponding controller. Then we briefly introduce the fully dynamic simulation model that is used to verify the learned forward models and the dynamic controller. Finally, we present details on the learning architecture together with results of the model and the closed-loop task-space controller.

## 2. Theory

Given a soft robot that can be kinematically modeled by the configuration-space *q* ∈ ℝ^*n*^, the task-space variable can be obtained by the kinematic transformation:

(1) $$\begin{array}{l} x=F\left(q\right) \end{array}$$

Where, *x* ∈ ℝ^*m*^ and *m* ≤ *n*. The task-space variable is typically the pose of the end-effector and is to be controlled. The configuration space is the set of independent variables that fully defines the state of the robot. The fully dynamic model of the soft robot can then be represented using the configuration-space variable as:

(2) $$\begin{array}{l} M\left(q\right)\ddot{q}+C\left(q,\dot{q}\right)\dot{q}+G\left(q\right)=\tau \end{array}$$

Here, *M*(*q*) represents the inertial properties, $C\left(q,\dot{q}\right)$ combines the coriolis, centrifugal and damping elements, *G*(*q*) represents the gravitational and stiffness effects and τ is the generalized force applied internally by the robot.

Soft robots typically have high damping values with low inertial properties. This is because they are commonly fabricated with viscoelastic materials with low material density. After the initial transient motion of a soft robot from rest (when $\dot{q}=0$), the first order term dominates the second order term [i.e., $C\left(q,\dot{q}\right)\dot{q}>>M\left(q\right)\ddot{q}$]. Hence, the second-order term can be ignored without sacrificing the accuracy of the model (Zajac, 1989). The dominant modeling error will occur during the initial transient motion (Strogatz, 2001). This transforms the second-order dynamical model (Equation 2) into:

(3) $$\begin{array}{l} C\left(q,\dot{q}\right)\dot{q}+G\left(q\right)=\tau \end{array}$$

After discretizing the equation, the dynamic model can now be represented through the mapping : (*q*_*i*_, τ_*i*_) → *q*_*i*+1_. Where, *q*_*i*_, *q*_*i*+1_ are the current and the next configuration of the soft robot, respectively. Correspondingly, this implies that a closed-loop dynamic controller would require only the zero-order state feedback (*q*) for control.

### 2.1. Controller Design

The obtained first-order dynamical Equation (3), the first-order configuration-space term can be replaced using the well known inverse kinematics mapping:

$$\begin{array}{l} ẋ=J\left(q\right)\dot{q} \end{array}$$

(4) $$\begin{array}{l} \dot{q}=J{\left(q\right)}^{†}ẋ \end{array}$$

Where, *J*(*q*) is Jacobian matrix and *J*(*q*)^†^ is any generalized inverse matrix. Note that we have ignored the null-space terms for brevity. Now the first-order dynamic equation can be reformulated as function of the task-space variables:

(5) $$\begin{array}{l} \tau =F\left(q,ẋ\right) \end{array}$$

This functional mapping can now be directly learning by a machine learning architecture. We can now transform the mapping and introduce the target task-space variable *x*^*d*^ as:

(6) $$\begin{array}{l} ẋ=x^{d}-x^{c} \end{array}$$

The operational space controller mapping now becomes:

(7) $$\begin{array}{l} \tau =F\left(q^{c},x^{d}\right) \end{array}$$

Here, (*q*^*c*^, *x*^*c*^) are the current configuration-space coordinates and the current task-space coordinates, respectively. For the special case when the cardinality of the configuration-space coordinate is the same as the cardinality of the task-space coordinate (mapping from *q* → *x* is bijective), the operational-space controller can be further simplified to a mapping: (*x*^*c*^, *x*^*d*^) → τ. A simple feedforward neural network can be used to learn this mapping (Figure 2). In this paper, we restrict our studies to this condition for simplicity. This allows us to test the learned controller by providing trajectories in the task-space without the need to solve the inverse kinematics problem. For redundant task-space controllers (non-bijective mappings), additional planning stages might be required to obtain the configuration-space trajectories. In other words, the control trajectory cannot be represented only in the task-space variables. In such a case, an augmented trajectory can be defined with the task-space variable along with a optimization routine to check for kinematic constraints as shown before in Thuruthel et al. (2017). Note that the trajectories are only zero-order task space variables and the feedback required for closed-loop control is also zero-order. This greatly reduces the requirement on the sensors, the effect of noise and the complexity of the trajectory planner.

## 3. Simulation Model

**Figure 1:**
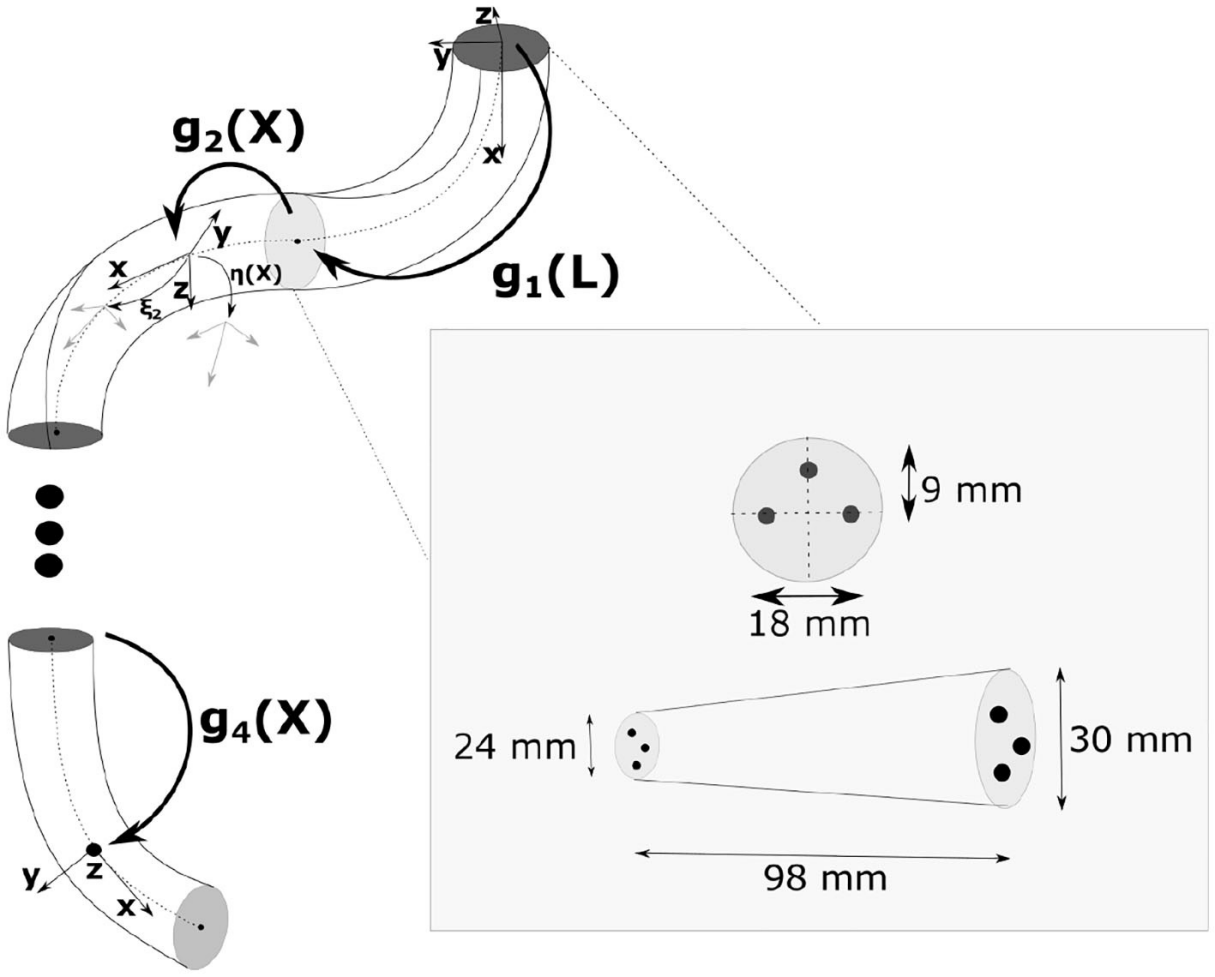
Schematic of the soft manipulator used for our studies. The manipulator is driven by three tendons arranged in the configuration shown above. Unless stated otherwise, the single section manipulator is used for the study.

The dynamic model is based on the Piece-wise Constant Strain (PCS) approach for soft-rigid multibody system of Renda and Seneviratne (2018) (see Figure 1). In the following, all the quantities are expressed in the local (body) coordinate frame if not specified otherwise. The superscript ′ and ˙ represent partial differentiation with respect to the space variable and time variables, respectively. The accent ^˜^ represents the usual isomorphism between a vector in ℝ^3^ and its corresponding skew-symmetric matrix in 𝔰𝔬(3).

### 3.1. Kinematics

The relative position and orientation of a soft body *i* with respect to its predecessor in the chain is defined as a curve ***g***_*i*_(·) : *X* ∈ [0, *L*_*i*_]↦***g***_*i*_(*X*) ∈ *SE*(3) with

$$g_{i}\left(X\right)=\left(\begin{array}{cc} {\text{R}}_{i} & {\text{u}}_{i}\\ 0^{T} & 1 \end{array}\right).$$

The continuous models of the position, velocity and acceleration of a soft body can be derived from the Cosserat rod theory, which gives (Boyer and Renda, 2016):

(8a) $$\begin{array}{l} g_{i}^{\prime }=g_{i}{\hat{\xi }}_{i}, \end{array}$$

(8b) $$\begin{array}{l} \eta_{i}^{\prime }={\dot{\xi }}_{i}-\text{a}{\text{d}}_{\xi_{i}}\eta_{i}, \end{array}$$

(8c) $$\begin{array}{l} {\dot{\eta }}_{i}^{\prime }={\ddot{\xi }}_{i}-\text{a}{\text{d}}_{{\dot{\xi }}_{i}}\eta_{i}-\text{a}{\text{d}}_{\xi_{i}}{\dot{\eta }}_{i}, \end{array}$$

where $\xi_{i}\left(X\right)\in {\mathbb{R}}^{6}$ defines the strain state, $\eta_{i}\left(X\right)\in {\mathbb{R}}^{6}$ is the cross-section velocity twist and $\text{a}{\text{d}}_{\left(\cdot \right)}\in {\mathbb{R}}^{6\times 6}$ is the adjoint operator of the Lie algebra (see Nomenclature). Going further into detail, we have

$${\hat{\xi }}_{i}\left(X\right)=\left(\begin{array}{cc} {\tilde{\text{k}}}_{i} & {\text{p}}_{i}\\ 0^{T} & 0 \end{array}\right)\in 𝔰𝔢\left(3\right),\xi_{i}\left(X\right)={\left({\text{k}}_{i}^{T},{{\text{p}}_{i}}^{T}\right)}^{T}\in {\mathbb{R}}^{6},$$

$${\hat{\eta }}_{i}\left(X\right)=\left(\begin{array}{cc} {\tilde{\text{w}}}_{i} & {\text{v}}_{i}\\ 0^{T} & 0 \end{array}\right)\in 𝔰𝔢\left(3\right),\eta_{i}\left(X\right)={\left({\text{w}}_{i}^{T},{\text{v}}_{i}^{T}\right)}^{T}\in {\mathbb{R}}^{6},$$

with ${\text{k}}_{i}\left(X\right)\in {\mathbb{R}}^{3}$ and ${\text{p}}_{i}\left(X\right)\in {\mathbb{R}}^{3}$ the angular and linear strain; and ${\text{w}}_{i}\left(X\right)\in {\mathbb{R}}^{3}$ and ${\text{v}}_{i}\left(X\right)\in {\mathbb{R}}^{3}$ the angular and linear velocity, respectively.

To model constrained rod, such as the Kirchhoff-Love case with angular strain only, the strain field is specified as:

$$\xi_{i}=B_{i}q_{i}+\xi_{i}^{*},$$

where $B_{i}\in {\mathbb{R}}^{6\times n_{i}}$ forms a basis for the allowed motion subspace, $q_{i}\in {\mathbb{R}}^{n_{i}}$ contains the values of the allowed strains and, $\xi_{i}^{*}\in {\mathbb{R}}^{6}$ is the reference twist modeling the reference shape.

Assuming piece-wise constant strains (Renda et al., 2016), Equations (8) can be analytically integrated using the matrix exponential method, leading to:

(9a) $$\begin{array}{l} g_{i}\left(X\right)=e^{X{\hat{\xi }}_{i}}, \end{array}$$

(9b) $$\begin{array}{l} \eta_{i}\left(X\right)={\text{Ad}}_{g_{i}}^{-1}\eta_{h}+{\text{Ad}}_{g_{i}}^{-1}{\text{T}}_{g_{i}}B_{i}{\dot{q}}_{i}, \end{array}$$

(9c) $$\begin{array}{l} {\dot{\eta }}_{i}\left(X\right)={\text{Ad}}_{g_{i}}^{-1}{\dot{\eta }}_{h}+{\text{Ad}}_{g_{i}}^{-1}\int_{0}^{X}\text{A}{\text{d}}_{g_{i}\left(s\right)}\text{a}{\text{d}}_{\eta_{i}\left(s\right)}dsB_{i}{\dot{q}}_{i}+\text{A}{\text{d}}_{g_{i}}^{-1}{\text{T}}_{g_{i}}B_{i}{\ddot{q}}_{i}, \end{array}$$

where $\text{A}{\text{d}}_{g_{i}}\left(X\right)\in {\mathbb{R}}^{6\times 6}$ is the Adjoint operator of *SE*(3), and ${\text{T}}_{g_{i}}\left(X\right)=\int_{0}^{X}e^{s\text{a}{\text{d}}_{\xi_{i}}}ds$ is the tangent operator of the exponential map, of which an analytic expression, derived from (Selig, 2007), is given in the Nomenclature.

Successive applications of the kinematics (Equation 9) for all the bodies of the system, yields to the definition of the geometric Jacobian $J_{i}\left(q,X\right)\in {\mathbb{R}}^{6\times n}$ and its derivative ${\dot{J}}_{i}\left(q,\dot{q},X\right)\in {\mathbb{R}}^{6\times n}$ (*n* being the total number of DOFs), which relates the generalized coordinate vector $q={\left[q_{1}^{T}q_{1}^{T}\;\cdots \;q_{N}^{T}\right]}^{T}\in {\mathbb{R}}^{n}$ (*N* being the total number of bodies) and the velocity twist **η**_*i*_(*X*), for each soft body *i*, as shown below.

(10a) ηi(X)=∑h=0iAdgh⋯gi-1TghBhq˙h=∑h=0iShiq˙h=Ji(q,X)q˙,

(10b) η˙i(X)=∑h=0iShiq¨h+Adgh⋯gi-1∫0XAdgh(s)adηh(s)dsBhq˙h   =∑h=0iShiq¨h+iS˙hq˙h=Ji(q,X)q¨+J˙i(q,q˙,X)q˙,

where the block elements of the *i*^*th*^ Jacobian ${\;}^{i}S_{\left(\cdot \right)}\in {\mathbb{R}}^{6\times n_{\left(\cdot \right)}}$ and its derivative ${\;}^{i}{\dot{S}}_{\left(\cdot \right)}\in {\mathbb{R}}^{6\times n_{\left(\cdot \right)}}$ have been defined. Note that the last three rows of Equation (10a) provide an analytical expression of the kinematics map required by Equation (4).

### 3.2. Dynamics

Once a Jacobian is found, the generalized dynamics of the system can be obtained by projecting the free dynamics of each soft body by virtue of the D'Alembert's principle. The free dynamic equation, with its boundary conditions, of a soft body is given by (Renda et al., 2018):

(11) $$\begin{array}{l} M_{i}{\dot{\eta }}_{i}+\text{a}{\text{d}}_{\eta_{i}}^{*}M_{i}\eta_{i}={\left(F_{i_{i}}-F_{a_{i}}\right)}^{\prime }+\text{a}{\text{d}}_{\xi_{i}}^{*}\left(F_{i_{i}}-F_{a_{i}}\right)+{\bar{F}}_{e_{i}},\\ \left(F_{i_{i}}-F_{a_{i}}\right)\left(0\right)=-F_{J_{i}},\left(F_{i_{i}}-F_{a_{i}}\right)\left(L_{i}\right)=-\text{A}{\text{d}}_{g_{ij}}^{*}F_{J_{j}}\text{;} \end{array}$$

where $M_{i}\left(X\right)=\text{diag}\left(J_{x_{i}},J_{y_{i}},J_{z_{i}},A_{i},A_{i},A_{i}\right)\rho_{i}\in {\mathbb{R}}^{6\times 6}$ is the screw inertia matrix of the cross-section (*J*_·_*i*__(*X*) being the second moment of area about the axis · and *A*_*i*_(*X*) the area of the cross-section); ${\bar{F}}_{e_{i}}\left(X\right)\in {\mathbb{R}}^{6}$ is the distributed external load; $F_{a_{i}}\left(X\right)\in {\mathbb{R}}^{6}$ is the internal wrench due to the distributed actuation (Renda et al., 2017); $F_{i_{i}}\left(X\right)\in {\mathbb{R}}^{6}$ is the internal wrench due to the elasticity of the soft body; $F_{J_{\left(\cdot \right)}}\in {\mathbb{R}}^{6}$ is the wrench transmitted across joint (·) and $\text{a}{\text{d}}_{\left(\cdot \right)}^{*}$ (respectively $\text{A}{\text{d}}_{\left(\cdot \right)}^{*}$) ∈ ℝ^6×6^ is the co-adjoint (respectively co-Adjoint) map of the Lie algebra (respectively Lie group) defined in Nomenclature. Regarding the internal elastic force, a linear viscoelastic constitutive model is usually chosen:

(12) $$\begin{array}{l} F_{i_{i}}\left(X\right)=\Sigma_{i}\left(\xi_{i}-\xi^{*}\right)+\Upsilon_{i}{\dot{\xi }}_{i}=\Sigma_{i}B_{i}q_{i}+\Upsilon_{i}B_{i}\dot{q_{i}}, \end{array}$$

where

$$\begin{array}{l} \Sigma_{i}\left(X\right)=\text{diag}\left(G_{i}J_{x_{i}},E_{i}J_{y_{i}},E_{i}J_{z_{i}},E_{i}A_{i},G_{i}A_{i},G_{i}A_{i}\right)\in {\mathbb{R}}^{6\times 6}\text{ and} \end{array}$$

$$\begin{array}{l} \Upsilon_{i}\left(X\right)=\text{diag}\left(J_{x_{i}},3J_{y_{i}},3J_{z_{i}},3A_{i},A_{i},A_{i}\right)\nu_{i}\in {\mathbb{R}}^{6\times 6} \end{array}$$

are the screw stiffness and viscosity matrix (*E*_*i*_ being the young modulus, *G*_*i*_ the shear modulus and ν_*i*_ the shear viscosity).

By Jacobian projection of the free dynamics (Equation 11), we obtain the generalized dynamics in its classical configuration-space form:

(13) $$\begin{array}{l} M\left(q\right)\ddot{q}+\left(C\left(q,\dot{q}\right)+D\right)\dot{q}+Kq=\tau \left(q\right)+F\left(q\right), \end{array}$$

where ***M*** ∈ ℝ^*n*×*n*^ is the generalized mass matrix, ***C*** ∈ ℝ^*n*×*n*^ is the generalized Coriolis matrix, ***D*** ∈ ℝ^*n*×*n*^ is the block-diagonal generalized damping matrix, ***K*** ∈ ℝ^*n*×*n*^ is the block-diagonal generalized stiffness matrix, ***F*** ∈ ℝ^*n*^ is the vector of generalized position-dependent external forces and **τ** ∈ ℝ^*n*^ is the vector of applied actuation forces. Note that the dynamic Equation (13) can be written in the form required by Equation (2), with $C\left(q,\dot{q}\right)=C\left(q,\dot{q}\right)+D$ and *G*(*q*) = ***Kq*** − ***F***(***q***). Going further into details, the coefficient matrices take the form:

(14a) $$\begin{array}{l} M\left(q\right)=\overset{N}{\underset{i=1}{\sum }}\int_{0}^{L_{i}}J_{i}^{T}M_{i}J_{i}dX, \end{array}$$

(14b) $$\begin{array}{l} C\left(q,\dot{q}\right)=\overset{N}{\underset{i=1}{\sum }}\int_{0}^{L_{i}}J_{i}^{T}\left(\text{a}{\text{d}}_{J_{i}\dot{q}}^{*}M_{i}J_{i}+M_{i}{\dot{J}}_{i}\right)dX \end{array}$$

(14c) $$\begin{array}{l} D=\text{diag}(B_{1}^{T}\int_{0}^{L_{1}}\Upsilon_{1}dXB_{1},B_{2}^{T}\int_{0}^{L_{2}}\Upsilon_{2}dXB_{2},\cdots ,\\ \;B_{N}^{T}\int_{0}^{L_{N}}\Upsilon_{N}dXB_{N}), \end{array}$$

(14d) $$\begin{array}{l} K=\text{diag}(B_{1}^{T}\int_{0}^{L_{1}}\Sigma_{1}dXB_{1},B_{2}^{T}\int_{0}^{L_{2}}\Sigma_{2}dXB_{2},\cdots ,\\ \;B_{N}^{T}\int_{0}^{L_{N}}\Sigma_{N}dXB_{N}), \end{array}$$

(14e) $$\begin{array}{l} \tau \left(q\right)=[{\left(B_{1}^{T}\int_{0}^{L_{1}}F_{a_{1}}dX\right)}^{T}{\left(B_{2}^{T}\int_{0}^{L_{2}}F_{a_{2}}dX\right)}^{T}\cdots \\ \;{{\left(B_{N}^{T}\int_{0}^{L_{N}}F_{a_{N}}dX\right)}^{T}]}^{T}, \end{array}$$

(14f) $$\begin{array}{l} F\left(q\right)=\overset{N}{\underset{i=1}{\sum }}\int_{0}^{L_{i}}J_{i}^{T}{\bar{F}}_{e_{i}}\text{.} \end{array}$$

It is worth noting here the different structure of the components of the generalized dynamics Equation (13). Similarly to the minimal Lagrangian models of traditional rigid robots, inertial loads are characterized by full coefficient matrices, as can be see from Equations (14a) and (14b), while damping and stiffness loads are characterized by block-diagonal coefficient matrices, as for Equations (14c) and (14d). This is in contrast with other modeling approaches that use absolute coordinates, such as Finite Elements, for which the opposite holds. Inertial coefficient matrices are block-diagonal while damping and stiffness coefficient matrices are full. Thus, neglecting inertial terms will be well suited for minimal Lagrangian models for soft robotic manipulator, such as the PCS approach.

## 4. Methods and Results

This section investigates two studies. First, we validate the accuracy of the learned first-order model with respect to the learned second-order model. Second, we perform simulated experiments to validate the accuracy of the proposed controller. All the tests are performed on the fully dynamic model described in section 3.

### 4.1. Dynamic Modeling

**Figure 2:**
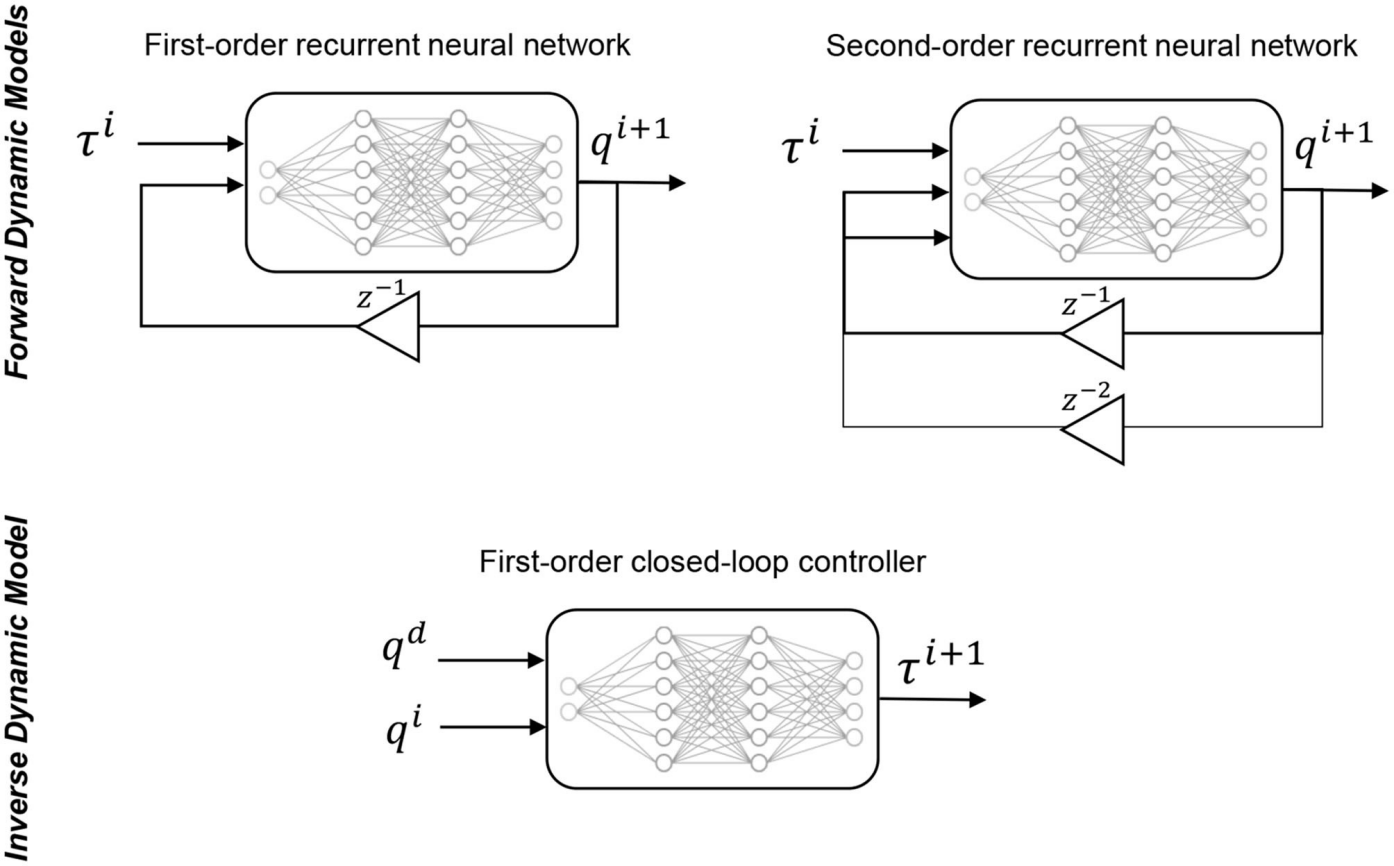
Modeling of the forward dynamics and the inverse dynamics controller. Note that for the non-redundant cases, the configuration-space (*q*) can be interchanged with the task-space variable (*x*), as done in this paper.

The learned models are derived using a kind of recurrent neural network called a nonlinear autoregressive exogenous (NARX) model (Billings, 2013). The NARX network is particularly suited for our study as it allows us to define the feedback horizon of the recurrent connection explicitly (Figure 2). In other words, we can ensure that the neural network receives only zeroth-order feedback for the first-order model and the appended first-order feedback for the second-order model. For the single section soft manipulator, the configuration-space (*q*) is equivalent to the task-space variable (*x*), which is defined as the three-dimensional position of the end-effector. We use a recurrent network with one hidden layer with a size of 40 for both the first-order and second-order model, for a fair comparison. The training parameters of the NARX network is given in Table 1.

**Table 1:** Parameters of the learned forward dynamic model.

**Parameter**	**Value**
Type	NARX network
Hidden layer size	40
No. of samples	7000
Training algorithm	Levenberg-Marquardt backpropagation
Training:Testing:Validation ratio	70:15:15
Stopping criterion	Validation set error
Maximum no. of epochs	100

**Figure 3:**
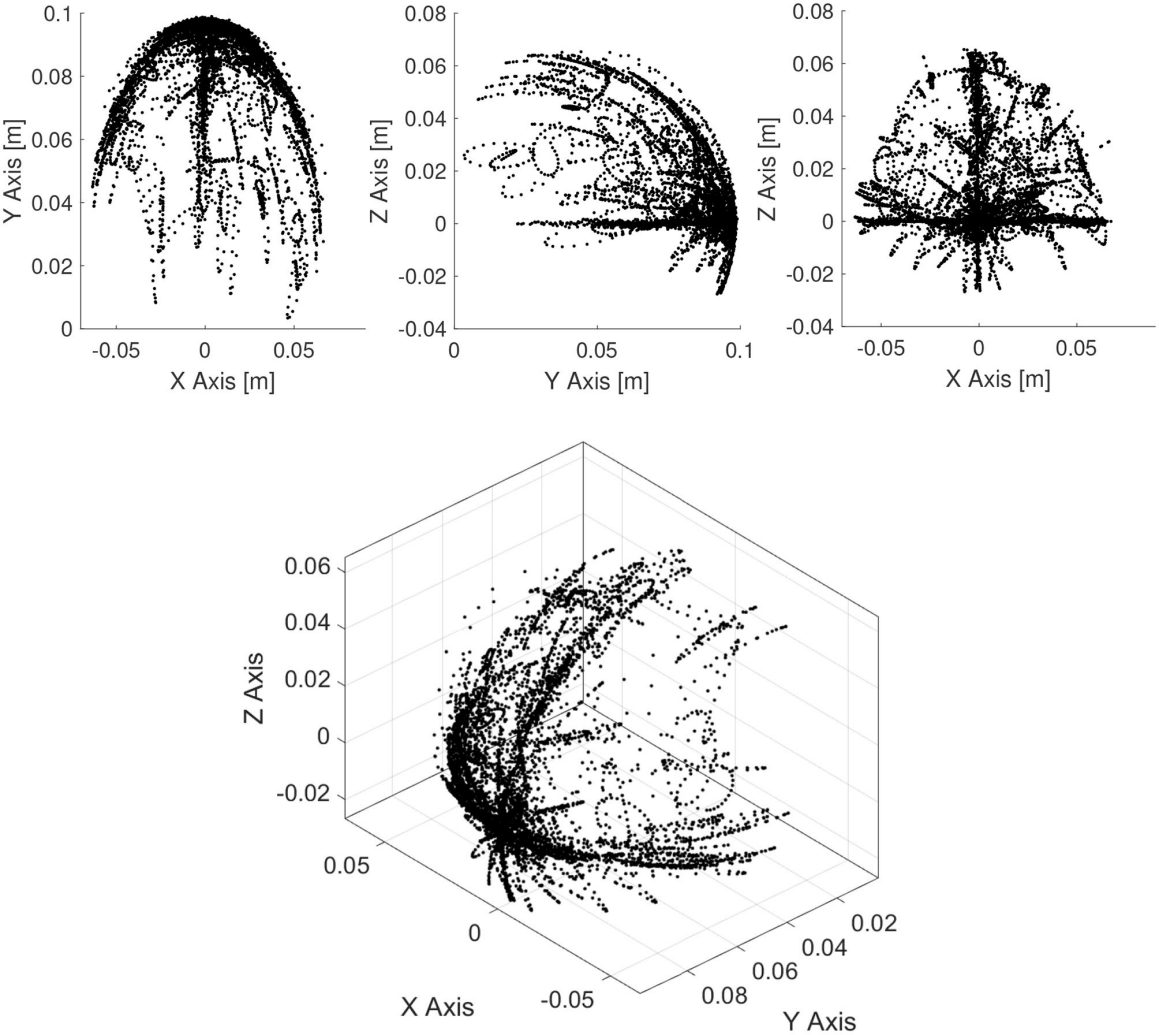
Dynamic workspace of the manipulator obtained by motor babbling. This is obtained by recording end-effector position of the manipulator when actuated by random continuous actuation signals.

Random actuation of the tendons are performed (motor babbling) for 70 s to obtain the samples for learning the forward model (Figure 3). Specifically, random actuation inputs are used to drive the manipulator and the corresponding actuator inputs and end-effector position is recorded over time. The same data samples can also be used for developing the closed-loop controller. Note that the dynamic workspace is concentrated along the direction of the three actuators. This is because tendons in tension have a strong attractor behavior. Hence, it will be easier to move along this direction. When testing our controller, careful measure is taken to ensure that our trajectories pass across this workspace regions.

**Figure 4:**
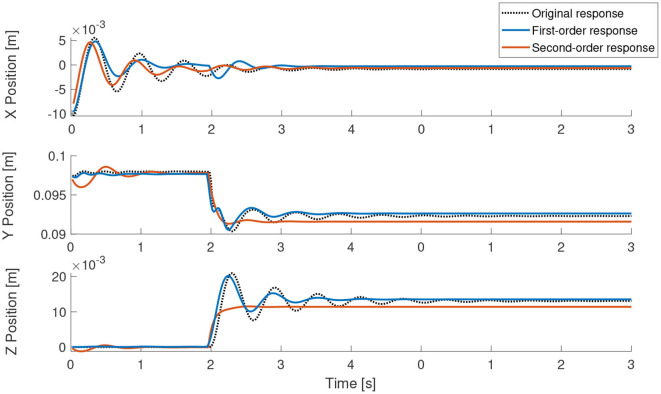
Step response of the learned models in comparison to the actual analytical model. The step signal is given to single actuator at time = 2 s.

**Figure 5:**
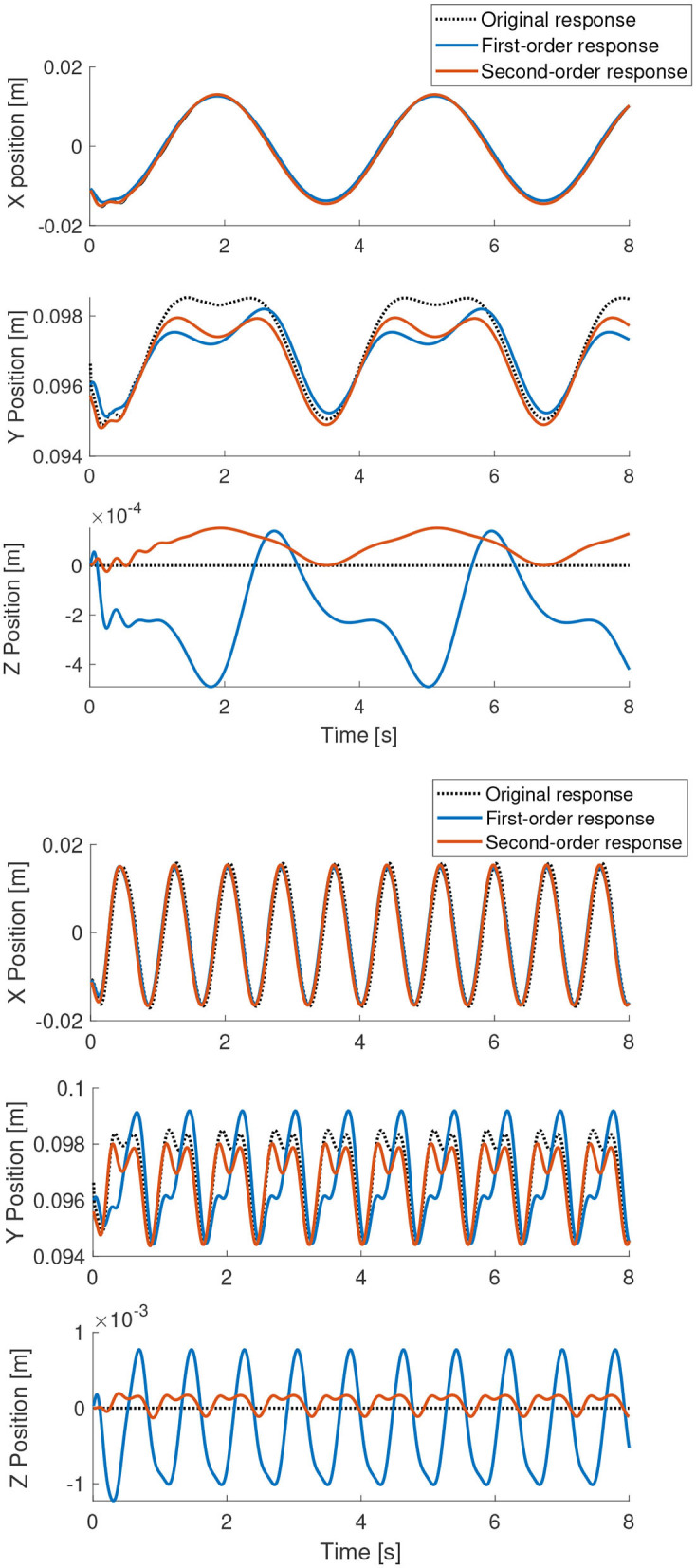
Periodic response of the learned models in comparison to the actual analytical model.

Figures 4, 5 show the performance of the learned model in comparison to the original cosserat model for a step and periodic response, respectively. Note the higher errors in the first-order model in the beginning of motion for the step response. Since the inertial effects are ignored, it is also visible that oscillations caused by overshoot is not found in the first-order model. However, the steady state error, with respect to the second-order model is relatively small. For the periodic excitation case, the difference between the first-order model and the second-order model is almost non-existent in the relevant coordinates. This is as expected since our approximations are more valid when the manipulator is in a non-stationary state.

In order to analyze the effects of the viscosity and the inertial effects on our modeling assumption, we further perform studies on the accuracy of the first-order model for varying material viscosity and density. As material density increases and the material viscosity decreases, the inertial effects become more and more dominant. Hence, one would expect the accuracy of the first-order model to decrease and the second-order model to remain constant. However, this is not necessarily the case as the training of second-order recurrent neural networks is more prone to instabilities (Pascanu et al., 2013).

**Figure 6:**
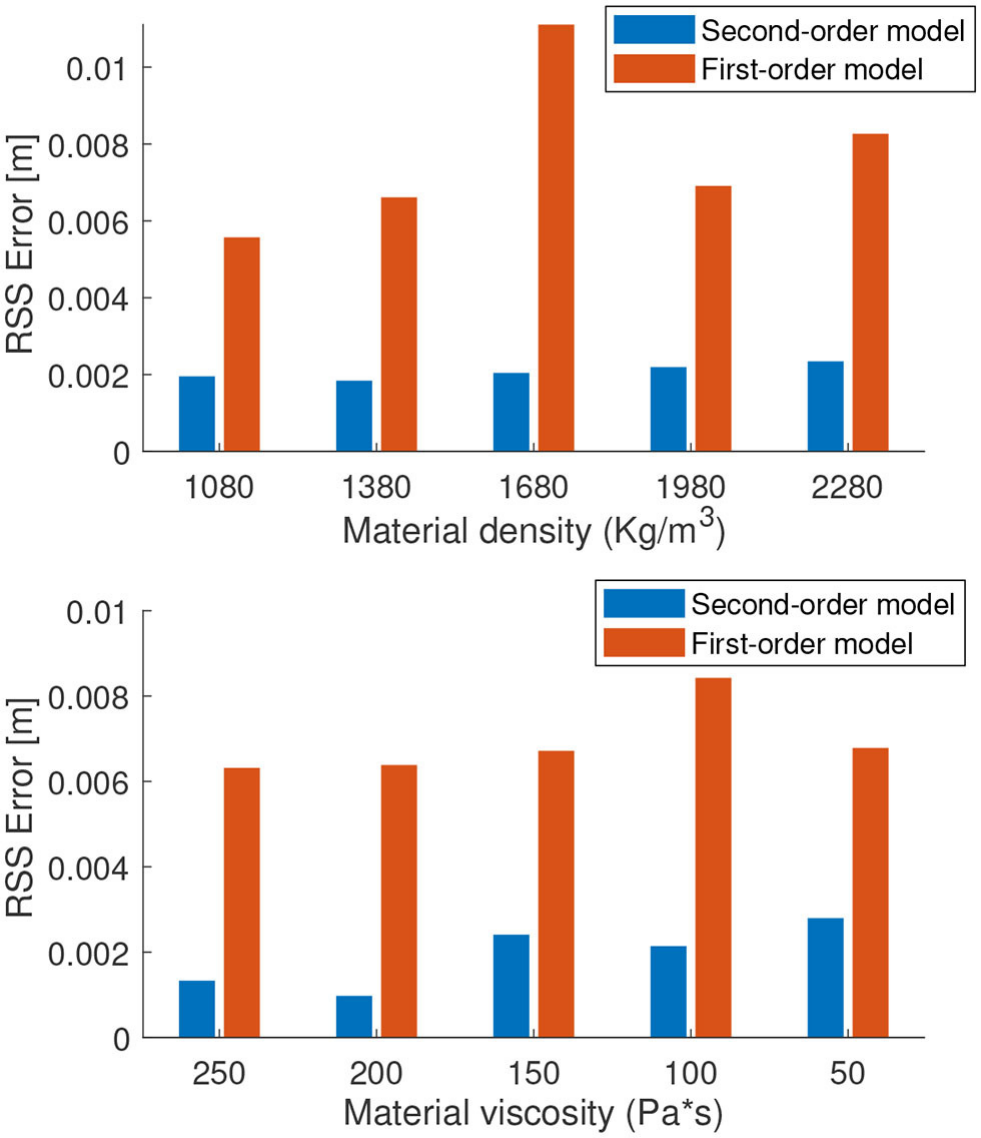
Root sum squared error of the learned model for varying dynamic properties of the manipulator.

Figure 6 shows how the root sum squared (RSS) error of the first-order model is affected when the material properties of the soft arm is changed in a way that weakens our main assumption. The material density is increased up to a factor of 2 and the material viscosity is reduced by a factor of 6. The motor babbling inputs and the neural network parameters are kept the same for the tests. It is clear from the prediction errors that the second-order model always performs better than the first-order model. However, the change in accuracy of both the models are not affected significantly by the change in material properties. Note that the initial parameters of the simulated cosserat model soft arm were obtained from real experiments on an Octopus-inspired soft manipulator, which was manufactured with silicone and driven by tendons (Renda et al., 2014).

**Figure 7:**
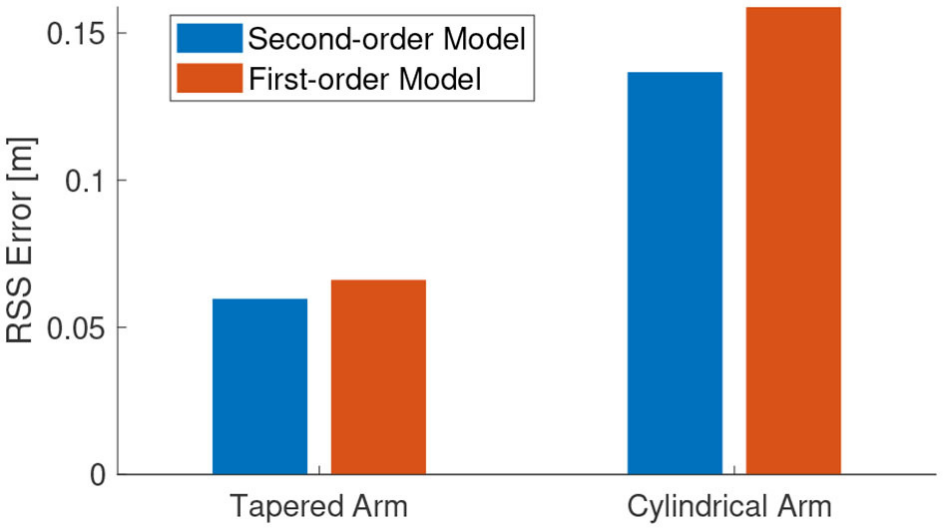
Root sum squared error of the learned model for the 4-section underactuated manipulator.

Increasing the length of the manipulator is another way to increase the inertial properties and weaken the first-order assumption. For this, we test the same methodology on a 4 section manipulator, however, actuated only on the first section. Each section has approximately the same length, with the total length of the manipulator adding to 418 mm. We test two designs, one with a tapered morphology and the other with a cylindrical morphology (higher inertial properties). The radius of the tapered morphology linearly reduces from 30 to 10 mm while the cylindrical morphology has a fixed diameter of 30 mm. For this test and the following controller results, the default parameters of the manipulator is used (i.e., with the material density of 1,080 kg/m^3^ and viscosity of 300 Pas; Renda et al., 2014). The results of our forward dynamics prediction on both the systems are shown in Figure 7. Contrary to our expectation, increasing the length of the manipulator did not affect the performance of the first-order model when compared to the second-order model. We believe this is because the inertial effects are still compensated by the medium (water) in which the manipulator is surrounded in. Our previous studies have shown that the dynamics of the manipulator becomes chaotic and hence unpredictable without a surrounding medium and when the length increases (Thuruthel et al., 2019). Based on our results, it can be deduced that this is more because of the first-order terms rather than the second-order terms. It could be because of the increased length, gravity, centripetal/centrifugal forces, higher DoFs etc. Note that this is a limitation of learning-based approaches as increased sensitivity to initial conditions decrease the stability of the training process and hence the performance of the model. The same applies to the parameter tuning process for analytical models. It could be concluded that it is a good practice to reduce the inertial effects of a soft-bodied system to improve the predictability of its dynamics, irrespective of the order of the model.

### 4.2. First-Order Dynamic Controller

The closed-loop task-space controller is derived by learning the operational-space dynamics mapping as described in section 2.1. As the mapping is not recursive, it can be learned using a simple feedforward neural network, as shown in Figure 2. For our non-redundant case, we can replace the configuration-space variable, *q*, with the task-space variable, *x*. The samples for learning the mapping is obtained through the same motor babbling process as described in the previous section. For training the controller the mapping is defined as: (*x*^*i*^, *x*^*i*+1^) → τ. When testing the controller, the next task-space coordinate, *x*^*i*+1^, is replaced by the desired task-space variable *x*^*d*^. The parameters of the neural network used for learning the first-order inverse dynamics mapping is given in Table 2.

**Table 2:** Parameters of the first-order inverse dynamics controller.

**Parameter**	**Value**
Type	Feedforward neural network
Hidden layer size	30
No. of samples	7,000
Training algorithm	Levenberg-Marquardt backpropagation
Training:Testing:Validation ratio	70:15:15
Stopping criterion	Validation set error
Maximum no. of epochs	100

Path planning is usually a complex problem in inverse dynamics based controllers, but as our inverse model is developed with only zeroth order state feedback, the development of the task-space trajectory is greatly simplified. Acceptable paths can easily be generated using the data points obtained from the workspace of the manipulator, which is obtained during the motor babbling phase. The desired paths can be generated by picking reachable points from the workspace and routing a path through them, ensuing that there is sufficient time for the manipulator to reach adjacent points. This can be easily done by fixing a cap on the maximum distance between adjacent task-space variables.

To test our controller, we generate randomized linear paths for the end-effector of the manipulator to follow. This is done by picking two random points from the robot workspace and linearly interpolating a trajectory between them and from the initial position of the end-effector. If the initial position of the end-effector is *p*^0^ and the two random points are *p*^1^ and *p*^2^, the generated path is from *p*^0^ → *p*^1^ → *p*^2^ → *p*^1^. Note that the intermediate points are not necessarily reachable by this naive approach. Accurate trajectories can be generated by searching for adjacent points in the workspace or by projecting the trajectory onto the workspace surface.

**Figure 8:**
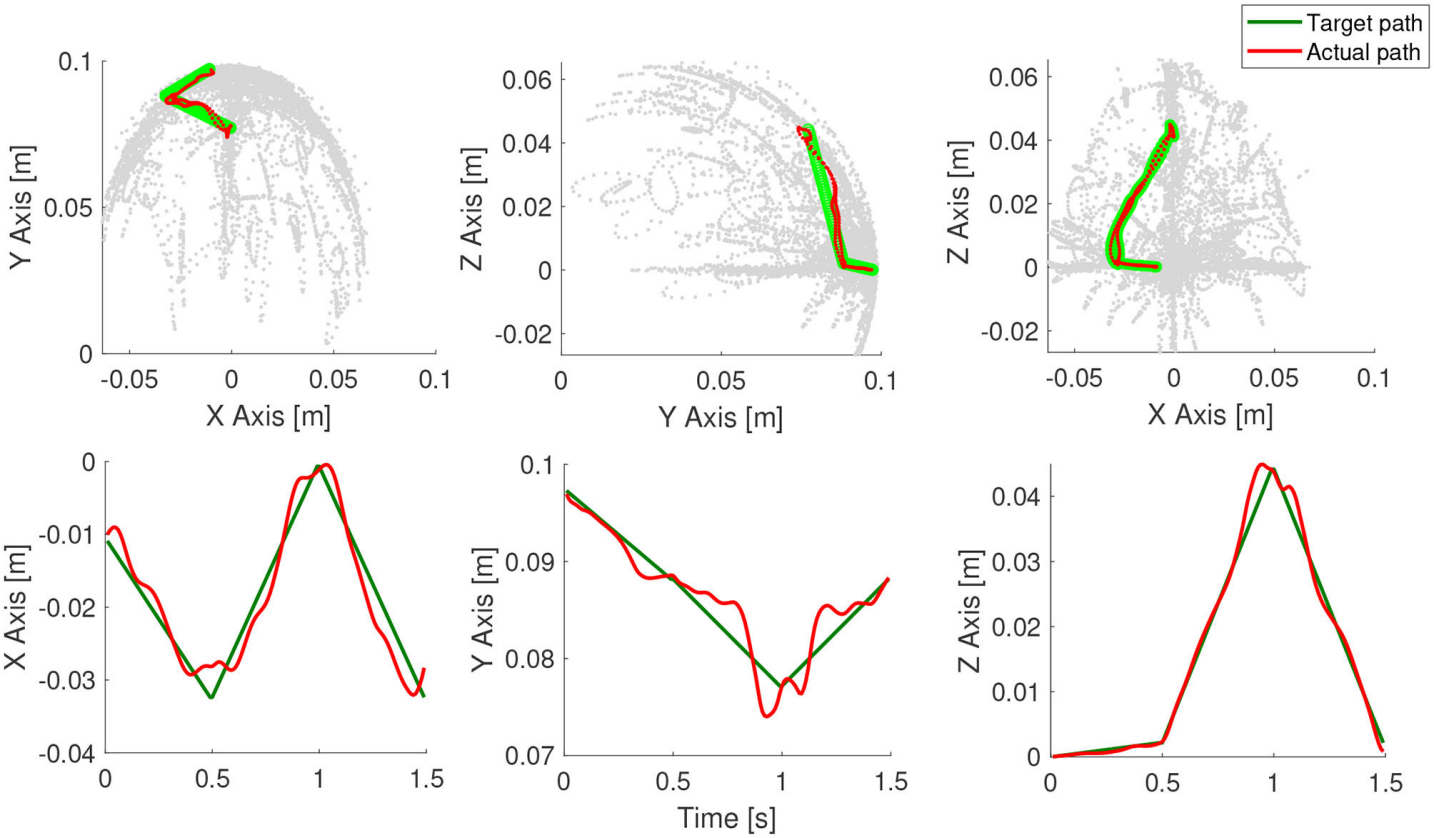
Controller results for a continuous path. The motion of the end-effector is shown here.

The results of the trajectory tracking is shown in Figure 8. The performance of the controller is excellent considering the fact that it is myopic with no step-ahead planning and the task-space trajectory being non-optimal. Due to the low computational cost in running the inverse-dynamic controller, we are able to run closed-loop task-space controller at a very high control frequency of 100 Hz. This will also allow the controller to compensate for any modeling errors incurred by the approximation.

**Figure 9:**
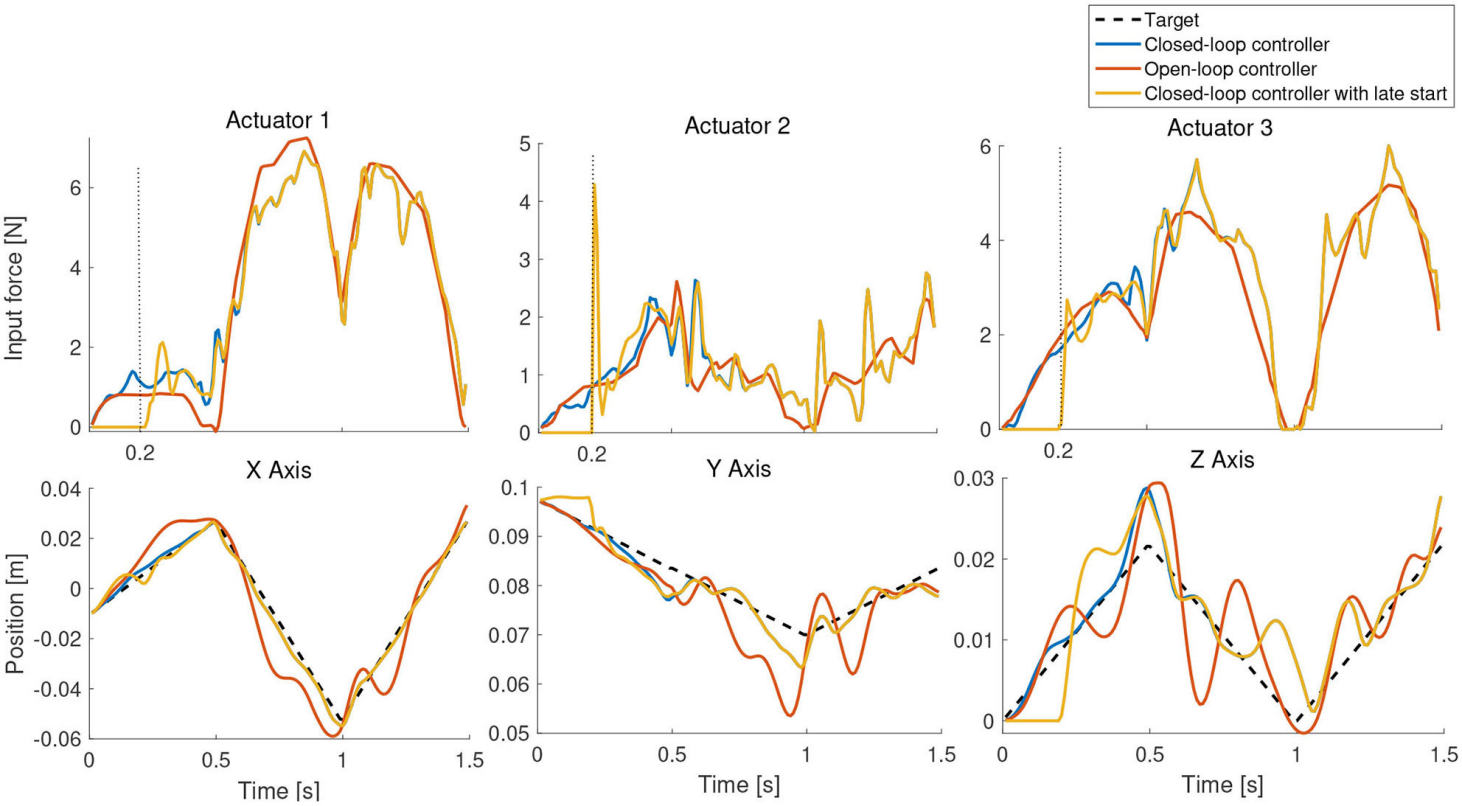
Testing the first-order controller in open-loop, closed-loop, and with a late start.

**Figure 10:**
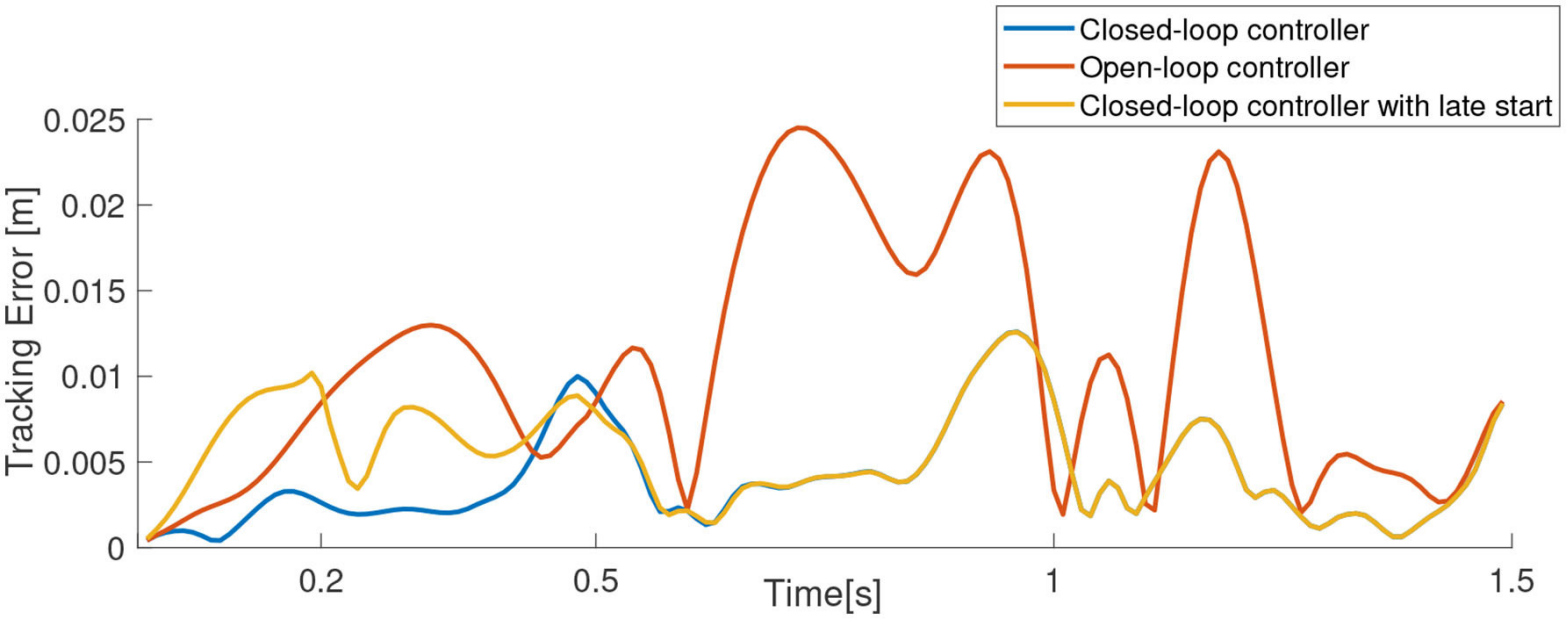
Tracking error for the scenario shown in Figure 9.

The *quality* of the task-space trajectory can be analyzed by running the controller in open-loop. This can be done by assuming the manipulator is able to reach each trajectory point perfectly and obtaining the best control action at each time step. The results of such a scenario is shown in Figure 9, along with the corresponding control inputs. As expected, the open-loop controller performs worse than the closed-loop controller even though there are no external disturbances in the simulation. The same scenario is repeated with the controller inactive for the first 0.2 s in Figure 9. As the desired targets are now farther from the current position of the manipulator, it is not necessary that the controller is able to follow the trajectory accurately. However, as evident from the results, the controller is able to recover and remarkably converge to the same solution as the original closed-loop controller. This also shows how important it is to close-the-loop, even at the cost of reduced model accuracy. The tracking error of the three tests described in this scenario is shown in Figure 10.

## 5. Conclusion

This paper presents and verifies a model simplifying assumption for soft robots. The core idea of the assumption is that soft robots, by definition, tend to have low inertial and high viscoelastic properties. This leads to dynamic behaviors which are well approximated by a first-order system, as typically observed in nature. We verify this assumption using a simulated fully dynamic model of an Octopus-like manipulator and a type of recurrent neural network called NARX network. Finally, we develop easy-to-develop closed-loop task-space dynamic controllers based on this assumption. Our results indicate that controllers developed on this assumption can compensate the errors in modeling accuracy with the increased control frequency. Our method makes path planning simpler for non-redundant cases. Moreover, the sensory requirements for closed-loop dynamic control is reduced because of the simplification. This is because the state feedback required for the controller is only the zeroth-order component. Our work also indicates that the additional modeling complexity that soft elements introduce can, to some extent, be reduced by designing low inertial highly visco-elastic soft robot designs.

Although we use machine learning tools to test our modeling assumption and develop our controller, the approach is equally suited for analytical approaches. In fact, ignoring the inertial elements would greatly simply the modeling and parameter estimation process involved in model-based control of soft robots. Not only can we reduce the states of the dynamical system, but we also avoid the problem of estimating and inverting the full mass matrix (see section 3.2). However, it must be kept in mind that first-order systems present numerical challenges in their implementation. Such problems are not found in a learning based approach and hence desirable in that respect. Typically, the first-order model leads to a stiff differential equation and requires specialized techniques for solving them. Interested readers are suggested to refer to Strogatz (2001) for further information. Future work involve extending the work to redundant systems.

## Data Availability Statement

The original contributions presented in the study are included in the article/supplementary material, further inquiries can be directed to the corresponding author/s.

## Author Contributions

All authors listed have made a substantial, direct and intellectual contribution to the work, and approved it for publication.

## Conflict of Interest

The authors declare that the research was conducted in the absence of any commercial or financial relationships that could be construed as a potential conflict of interest.

## Nomenclature

[ad_***ξ***, ***η***_] $\left(X\right)=\left(\begin{array}{cc} \tilde{\text{k}},\tilde{\text{w}} & 0_{3\times 3}\\ \tilde{\text{q}},\tilde{\text{v}} & \tilde{\text{k}},\tilde{\text{w}} \end{array}\right)\in {\mathbb{R}}^{6\times 6}$. adjoint representation of the strain twist vector in *SE*(3).

[$\text{a}{\text{d}}_{\xi ,\eta }^{*}$] $\left(X\right)=\left(\begin{array}{cc} \tilde{\text{k}},\tilde{\text{w}} & \tilde{\text{q}},\tilde{\text{v}}\\ 0_{3\times 3} & \tilde{\text{k}},\tilde{\text{w}} \end{array}\right)\in {\mathbb{R}}^{6\times 6}$. coadjoint representation of the strain twist vector in *SE*(3).

[Ad_***g***_] $\left(X\right)=\left(\begin{array}{cc} R & 0_{3\times 3}\\ \tilde{\text{u}}R & R \end{array}\right)\in {\mathbb{R}}^{6\times 6}$. Adjoint representation of *SE*(3).

[$\text{A}{\text{d}}_{g}^{*}$] $\left(X\right)=\left(\begin{array}{cc} R & \tilde{\text{u}}R\\ 0_{3\times 3} & R \end{array}\right)\in {\mathbb{R}}^{6\times 6}$. coAdjoint representation of *SE*(3).

[$e^{X\hat{\xi }}$] $=I_{4}+X\hat{\xi }+\frac{1}{\theta^{2}}\left(1-\cos\left(X\theta \right)\right){\hat{\xi }}^{2}+\frac{1}{\theta^{3}}\left(X\theta -\sin\left(X\theta \right)\right){\hat{\xi }}^{3}$. Exponential map in *SE*(3) with θ^2^ = k^*T*^k.

[T_***g***_] $\left(X\right)=XI_{6}+\frac{1}{2\theta^{2}}\left(4-4\cos\left(X\theta \right)-X\theta \sin\left(X\theta \right)\right)\text{ a}{\text{d}}_{\xi }+\frac{1}{2\theta^{3}}\left(4X\theta -5\sin\left(X\theta \right)+X\theta \cos\left(X\theta \right)\right)\text{ a}{\text{d}}_{\xi }^{2}+\frac{1}{2\theta^{4}}\left(2-2\cos\left(X\theta \right)-X\theta \sin\left(X\theta \right)\right)\text{ a}{\text{d}}_{\xi }^{3}+\frac{1}{2\theta^{5}}\left(2X\theta -3\sin\left(X\theta \right)+X\theta \cos\left(X\theta \right)\right)\text{ a}{\text{d}}_{\xi }^{4}$. Analytic expression of the tangent operator of the exponential map in *SE*(3).
